# Spatial variability in factors influencing maternal health service use in Jimma Zone, Ethiopia: a geographically-weighted regression analysis

**DOI:** 10.1186/s12913-021-06379-3

**Published:** 2021-05-12

**Authors:** Jaameeta Kurji, Charles Thickstun, Gebeyehu Bulcha, Monica Taljaard, Ziqi Li, Manisha A. Kulkarni

**Affiliations:** 1 School of Epidemiology and Public Health, University of Ottawa, 600 Peter Morand Crescent, Ottawa, Ontario K1G 5Z3 Canada; 2 Jimma Zone Health Office, Jimma town, Jimma Zone, Oromia Region Ethiopia; 3 Ottawa Hospital Research Institute, Ottawa, Canada; 4 Department of Geography & Geographic Information Science, University of Illinois, Urbana, USA

**Keywords:** Geographically weighted regressions, Spatial heterogeneity, Ethiopia, Maternal health services, Responsive health systems, Sub-national data, Equity, Local policy

## Abstract

**Background:**

Persisting within-country disparities in maternal health service access are significant barriers to attaining the Sustainable Development Goals aimed at reducing inequalities and ensuring good health for all. Sub-national decision-makers mandated to deliver health services play a central role in advancing equity but require appropriate evidence to craft effective responses. We use spatial analyses to identify locally-relevant barriers to access using sub-national data from rural areas in Jimma Zone, Ethiopia.

**Methods:**

Cross-sectional data from 3727 households, in three districts, collected at baseline in a cluster randomized controlled trial were analysed using geographically-weighted regressions. These models help to quantify associations within women’s proximal contexts by generating local parameter estimates. Data subsets, representing an empirically-identified scale for neighbourhood, were used. Local associations between outcomes (antenatal, delivery, and postnatal care use) and potential explanatory factors at individual-level (ex: health information source), interpersonal-level (ex: companion support availability) and health service-levels (ex: nearby health facility type) were modelled. Statistically significant local odds ratios were mapped to demonstrate how relevance and magnitude of associations between various explanatory factors and service outcomes change depending on locality.

**Results:**

Significant spatial variability in relationships between all services and their explanatory factors (*p* < 0.001) was detected, apart from the association between delivery care and women’s decision-making involvement (*p* = 0.124). Local models helped to pinpoint factors, such as danger sign awareness, that were relevant for some localities but not others. Among factors with more widespread influence, such as that of prior service use, variation in estimate magnitudes between localities was uncovered. Prominence of factors also differed between services; companion support, for example, had wider influence for delivery than postnatal care. No significant local associations with postnatal care use were detected for some factors, including wealth and decision involvement, at the selected neighbourhood scale.

**Conclusions:**

Spatial variability in service use associations means that the relative importance of explanatory factors changes with locality. These differences have important implications for the design of equity-oriented and responsive health systems. Reductions in within-country disparities are also unlikely if uniform solutions are applied to heterogeneous contexts. Multi-scale models, accommodating factor-specific neighbourhood scaling, may help to improve estimated local associations.

**Supplementary Information:**

The online version contains supplementary material available at 10.1186/s12913-021-06379-3.

## Background

Policies to reduce maternal and infant mortality often target improving utilization of essential maternal health services including antenatal, delivery, and postnatal care (PNC). Linking women and their newborns to care provides opportunities to detect and manage potential complications early on [1]. Reported use of these essential services has been steadily increasing in low- and middle-income countries over the last few decades [2, 3]. However, use of delivery and postnatal services has generally been lagging behind antenatal care (ANC) [4, 5].

In Ethiopia, women reported 27% ANC, 5% delivery care and 2% PNC use in 2000 [6]; by 2019, national levels had reached 74, 48, 34% respectively [7]. However, substantial within-country variation was noted with several regions recording utilization levels below the national average in all three services. In order to meet Sustainable Development Goal targets 3.1 and 3.2, which tackle maternal and child mortality [8], variation in service use at sub-national levels needs to be addressed to ensure equitable progress. More importantly, understanding how local contexts change the prominence of factors affecting use is necessary to create policy strategies that are responsive to local needs and make effective use of resources.

A range of individual characteristics (such as attitude towards delivery care), inter-personal factors (like women’s involvement in decision-making), and household factors (such as wealth or parity have been reported to influence maternal health service use [9–14]. However, these associations are typically quantified using regression models that assume relationships are constant across the entire study area (stationary relationships). Estimates generated from these “global models” represent averages that can mask important variation between localities [15]. Moreover, the presence of spatial dependence (where locations exhibit values that are similar to neighbouring locations) leads to spatially autocorrelated residuals that would violate the assumption of independent and identical error terms on which global models operate [16].

Exploratory work from three districts in Jimma Zone, Ethiopia, found evidence for spatial autocorrelation in the use of all three essential maternal health services [17]. Clusters with either higher (hotspots) or lower (cold spots) than expected levels of service use were identified at primary health care unit (PHCU)-, *kebele-* (village) and *sub-kebele* levels. This variability in service utilization may be indicative of underlying differences between localities in both the types of factors that are important for service use as well as the magnitude of associations. In fact, the impact of community influences on maternal health service use has also been previously discussed in qualitative studies [18, 19]. Differences in neighbourhood wealth levels, norms around permission to visit health facilities, community views on giving birth at home or perceptions about quality of care developed through experiences of social network members can all contribute to regional variability in service use [18, 19]. Contrasts in terrain and road access are also possible across different regions. If spatial mechanisms, where relationships depend on locality, have a role to play in observed patterns of service use, this needs to be appropriately explored to identify underlying factors.

The objective of this analysis, therefore, is to characterize non-stationarity in associations between explanatory factors and use of essential maternal health service use in Jimma Zone using geographically weighted regressions models.

## Methods

### Study setting

Ethiopia is situated in north-eastern Africa and has a total land area of over one million square kilometres [20]. Altitudes range between 110 below sea level around the Denakil Depression to more than 4600 m above sea level in the Simien Mountain ranges [20]. Jimma Zone is located in the southwest of the country within Oromia region. Administratively, Ethiopia has nine regional states which are further divided into *woredas* (districts) that comprise several *kebeles* (villages). The lowest level of the tiered health system operates at *woreda* level where PHCUs exist. PHCUs comprise a health centre that typically offers ANC, PNC, and basic emergency obstetric services. Each PHCU also has several community-based health posts that serve between 3000 and 5000 people and are staffed by health extension workers (HEWs) responsible for health promotion and preventive care in the community [21]. The Jimma University and Shenen Gibe general hospitals, which both provide comprehensive emergency obstetric care, are located in Jimma town.

This study was conducted in Gomma, Kersa and Seka Chekorsa districts. While agriculture dominates income generation in all three study districts, Gomma has substantial coffee production which is an important income source for many households [22]. Altitude ranges between 1500 and 2700 m across the three districts. In 2016, there were approximately 56,700 households in Gomma, 52,300 households in Seka Chekorsa, and 43,900 households in Kersa district [23].

The data for this study were obtained from a cross-sectional, baseline household survey conducted as part of a cluster-randomized controlled trial to evaluate the effectiveness of upgraded maternity waiting homes and local leader training in improving access to maternal health services. Baseline data was collected between October 2016 and January 2017. Details about the trial are available in the published protocol [24]. Briefly, we randomly assigned 24 PHCUs (clusters) in a 1:1:1 ratio to one of the two intervention arms or to usual care. Repeat cross-sectional surveys at baseline (prior to intervention roll-out) and endline were used to collect data from random samples of 160 women per cluster during each survey round. Women were eligible if they reported a pregnancy outcome (livebirth, stillbirth, miscarriage or abortion) up to 12 months prior to each survey. The number of women interviewed were 3784 (98.5% response rate) at baseline.

**Fig. 1:**
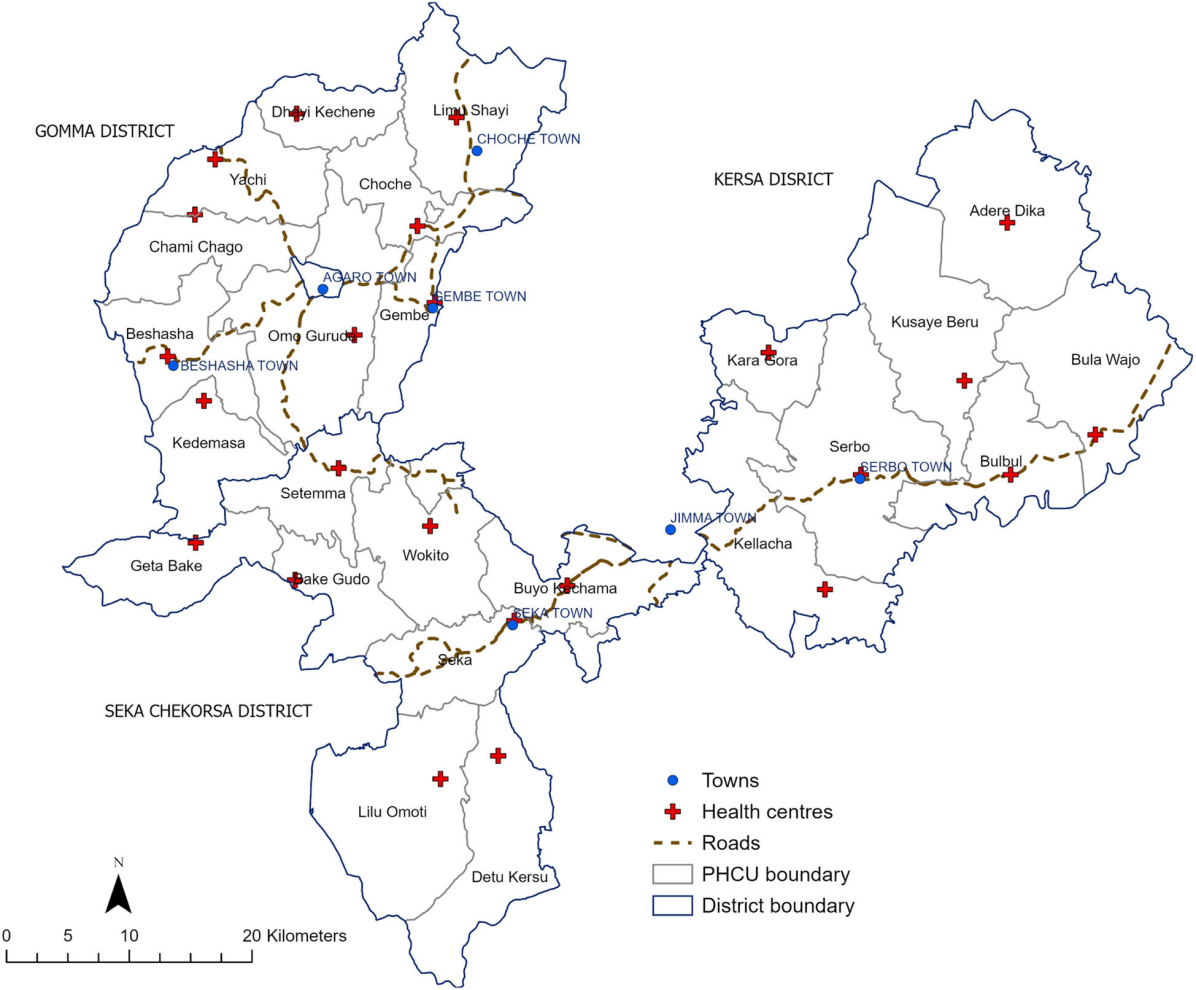
Map of the study area showing locations of health centres in PHCUs, main towns, roads, PHCU and district boundaries created in ArcGIS Pro

Data and GPS locations (collected using tablet computers) were available for 3727 households (98% of enrolled households) from 96 *kebeles*. GPS locations were also collected for all 24 health centres. Locations were mapped using ArcGIS Pro (ESRI, Redlands, USA) and projected into Adindan UTM Zone 37 N prior to analysis. Administrative boundary, town location and road network data were obtained from the Jimma Zone Health Office. A map of the study area created in ArcGIS Pro is included in Fig. 1.

### Variables of interest

Women’s self-reported utilization of ANC, delivery care, and PNC services for their last pregnancy/birth were the main outcomes of interest. These were constructed as binary variables at the individual woman level. ANC use was defined as whether or not women reported at least four ANC contacts with service providers during their last pregnancy at a health post, health centre or hospital, where these services are normally provided. Delivery care use was defined as whether or not women reported giving birth to their last child at a health centre or hospital, where basic emergency obstetric care is usually available. PNC use was defined as whether or not women reported receiving a check from a health worker at least 1 h after giving birth to their last child. The 1 h cut-off was used to distinguish between intrapartum and postpartum care which has been reported to be conflated by women [25]. Levels of service use among women in the baseline survey were 47% for at least four ANC contacts and, 49% for delivery care and 39% for PNC [26].

Candidate explanatory variables hypothesized to affect service use were identified based on the literature [9–14] and field experience. These were broadly categorized into individual woman characteristics, interpersonal or household elements and, health system-related considerations (Additional File 1: conceptual model). Factors hypothesized to be associated with all three services were: woman’s education, health information source, danger sign awareness, prior service use, household wealth, woman’s involvement in decision making, parity, home visits by HEWs and type of nearby health facility. Additionally, for ANC and delivery care use, perceived need for delivery care services, birth preparedness were considered important; availability of companion support was expected to be more relevant for delivery and postnatal care. Mode of delivery was expected to be an important factor associated with PNC use.

Frequencies and percentages (for categorical variables) and summary statistics (such as mean and standard deviation) for the continuous variable (parity) were generated to describe the study population.

Health system factors such as quality of care are important, but since they are common across entire PHCUs they are unlikely to exhibit sufficient variability at the local level required for geographically weighted regression (GWR) models. Distance between households and health centres was also not included in the models as it could confound GWR results which employ distance-based analyses [27]. Finally, husband characteristics, such as education level, and risk perceptions around complications among both women and their husbands were not included in the models since missing data reduced available sample size and could introduce selection bias. Definitions for explanatory variables hypothesized to be important factors influencing service use are provided in Additional File 2.

### Global regression models and presence of spatial dependencies

Before exploring spatial variation in relationships, the presence of spatial dependency needs to be established. This is usually done by testing the residuals from global models for the presence of spatial autocorrelation. Random effects multivariable logistic regression was conducted for each outcome (i.e., ANC, delivery care, PNC) with relevant candidate explanatory factors specified as fixed effects and PHCUs specified as random effects to account for intracluster correlation. Analysis was conducted in Stata version 15 (StataCorp, College Station, USA) and odds ratios with corresponding 95% confidence intervals were reported for each explanatory variable. These global estimates represent the mean values across the entire study area.

Deviance residuals were then generated and tested for the presence of spatial autocorrelation using Global Moran’s I spatial statistic in ArcGIS Pro. The Moran’s I index generally ranges from − 1 to 1; positive indices imply a clustering of similar values while negative indices are suggestive of more dispersed patterns [28]. A statistically significant Moran’s I index would imply that a spatial correlation structure exists in the residuals that needs to be explored using models that can integrate this spatial dependence.

### Exploring locally varying relationships using geographically weighted regression models

Geographically weighted regressions are an extension of conventional regression models that permit the estimation of coefficients for each location of interest (local estimates). In this way they can quantify non-stationary relationships which vary across space. The process is rooted in the first law of geography which asserts that neighbouring objects are more closely related than more distant objects [29]. As shown below, parameter estimates for *k* independent variables are estimated for each location *i*, in this case households, specified by coordinates (*u*_*i*_*, v*_*i*_) [15]:

$$ \log \left(\frac{\mathrm{E}\left({\mathrm{y}}_{\mathrm{i}}\right)}{1-\mathrm{E}\left({\mathrm{y}}_{\mathrm{i}}\right)}\right)={\beta}_0\left({\mathrm{u}}_{\mathrm{i}},{\mathrm{v}}_{\mathrm{i}}\right)+{\sum}_k{\beta}_{\mathrm{k}}\left({\mathrm{u}}_{\mathrm{i}},{\mathrm{v}}_{\mathrm{i}}\right){\mathrm{x}}_{\mathrm{i}\mathrm{k}}+{\mathrm{e}}_i $$

The “local” parameter estimates are generated using subsets of data points that are considered to be neighbours of household *i*. Neighbourhood is defined using spatial kernels and bandwidths parameters. The kernel is a proximity weighting function while the bandwidth is a measure of the distance decay in the kernel [15]. Whereas global models would assign the same weight to all household data points, kernels used in GWRs assign more weight to nearby households.

GWR analysis was conducted in MGWR 2.2 [30]. An adaptive, bi-square function, shown below, was used as the kernel, where weights assigned to neighbouring households (j) decrease according to a near-Gaussian curve up to the bandwidth (b), after which they are assigned a weight of zero [15].

$$ {\mathrm{w}}_{\mathrm{ij}}={\left[1-{\left({\mathrm{d}}_{\mathrm{ij}}/\mathrm{b}\right)}^2\right]}^2\ \mathrm{if}\kern0.5em j\kern0.5em \mathrm{is}\ \mathrm{an}\ \mathrm{nth}\ \mathrm{nearest}\ \mathrm{neighbour} $$

$$ {\mathrm{d}}_{\mathrm{ij}}\kern0.5em \mathrm{is}\ \mathrm{the}\ \mathrm{distance}\ \mathrm{between}\kern0.5em i\kern0.5em \mathrm{and}\kern0.5em j $$

In this way, the weights determine the level of contribution each household makes to the local model calibration process [15]. An adaptive rather than fixed kernel was selected to ensure that all local model calibration subsets had an adequate number of households. Fixed kernels can result in local estimates with large standard errors in areas with fewer data points when data points are not evenly distributed across the study area [15].

Optimization procedures are recommended when selecting bandwidths [15] as GWR estimates are sensitive to bandwidth choice. Large bandwidths may be unable to capture local variation and can return coefficients close to global model estimates. On the other hand, small bandwidths can result in high variability as coefficients are overly dependent on nearby points [15]. The Golden Section Search optimization technique was used to identify the optimal bandwidth that minimized the corrected Akaike Information Criterion (AICc) [15]. Optimal bandwidths were determined to be 927 households (872–2304) for ANC, 1459 households (1247–1573) for delivery care and 1560 households (1443–2296) for PNC.

### Model diagnostics and selection of the final local model

The potential for multicollinearity between local coefficients has been previously described as a concern for GWRs [31]. However, subsequent simulation studies with large sample sizes (≥ 1000) have demonstrated that GWRs estimates are not affected even in the presence of moderate global collinearity [32]. The results of diagnostic tests to check for multicollinearity in local parameter estimates, including condition numbers, local variance inflation factors (VIFs) and variance decomposition proportions (VDPs) were inspected nonetheless. Condition numbers greater than 30, VIFs greater than 10 and VDPs greater than 0.5 generally indicate a strong presence of multicollinearity [33–35]. Education and nearby health facility were, thus, removed from ANC and PNC models respectively. The final combination of explanatory factors retained in the local models had no evidence of local multicollinearity.

A test for spatial variability was also run to identify which relationships were significantly non-stationary. The null hypothesis of this test is that the associations of the explanatory factor with the outcome is globally fixed; a Monte Carlo approach is used to generate an experimental distribution of the variance of local parameters for each explanatory factor to which the actual variance is then compared [15].

Statistically significant estimates identified using adjusted *p*-values from the pseudo t-tests were exponentiated and mapped as odds ratios to visualize non-stationary relationships. Under pseudo t-tests, t-values are computed as a ratio between the estimate and its standard deviation and compared to a critical t-value that is adjusted for multiple testing using a Bonferroni-style correction adapted for GWRs [15, 36]. The adjusted margin of error (α) was 0.005 for ANC, 0.009 for delivery care and 0.010 for PNC. Significant estimates were mapped in colour using natural breaks with darker shades indicating higher magnitude, while non-significant estimates were mapped in grey. Only qualitative comparisons can be made between maps for the three services as association estimates are classified differently for the same explanatory factors.

The relative performance between the global and local models was compared by inspecting the respective AICc for each model [34]. The lower AICc obtained for local models compared to global models indicated that the former has the “best fit to the data” [15] and, were therefore, more desirable options. Finally, the residuals from the GWR models were tested using Global Moran’s I to see if there were any remaining spatial autocorrelation structures.

## Results

### Characteristics of the study population

Most women in the study area were housewives and about 45% had completed some level of education (Table 1). About half the women identified nurses as sources for birth-related information. While the majority of women were aware of at least one danger sign associated with pregnancy as well as birth, almost 60% were unaware of postpartum danger signs. In terms of prior maternal health service use, close to 60% of women had used ANC services for past pregnancies, but only half as many reported prior delivery care use. Almost all women felt delivery care was necessary for all women (94%) and most had companion support available (78%), were involved in decisions about delivery site (78%) and prepared for birth (68%).

**Table 1:** Frequencies, percentages, district- and PHCU-level ranges of explanatory factors

Characteristic	Frequency (*n* = 3727) (%)		District-level range (*n* = 3) (%)	PHCU-level range (*n* = 24) (%)
**Individual factors**				
Education level				
None	2068	(55.5)	47–68	31–73
Primary/secondary/higher	1659	(44.5)	32–53	27–69
Occupation				
Housewife	2884	(77.4)	76–80	67–90
Formal occupation	843	(22.6)	21–24	10–33
Danger sign awareness				
Aware of pregnancy danger signs	2784	(74.7)	74–75	60–93
Aware of delivery danger signs	2959	(79.4)	78–81	67–92
Aware of postpartum signs	1548	(41.5)	40–43	29–61
Nurse as information source				
Health-related information^a^	1543	(41.4)	37–47	15–53
Birth-related information	1874	(50.3)	45–56	18–66
Service use				
History of ANC use^a^	2070	(56.1)	50–65	21–83
ANC use for last child	1756	(47.1)	38–55	26–62
History of delivery care use^a^	1165	(31.6)	21–43	11–51
Delivery care use for last child	1835	(49.0)	35–64	19–72
Attitude towards delivery care				
Unnecessary for experienced women	239	(6.5)	6–7	1–16
Assisted delivery mode^a^	187	(5.0)	4–6	1–11
**Household or inter-personal factors**				
Wealthiest household group	1184	(31.8)	15–53	6–68
Companion support available	2907	(78.0)	70–86	5–53
Involved in decision making				
About delivery site	2916	(78.2)	76–81	54–84
Health-related issues	2656	(71.3)	67–75	59–91
Pregnancy planned	2438	(66.1)	56–73	42–81
Engaged in birth preparedness and planning	2520	(67.6)	61–72	16–52
**Health system factors**				
Home visit by HEW	1251	(33.6)	23–39	7–49
Nearby health facility type/level				
Hospital/health centre	1751	(47.5)	42–54	28–74

^a^Denominators differ: *Nurse as source of health* information, data available for *n* = 3721 (99.8%) women only. History *of ANC* use, data available for *n* = 3688 (98.7%) women only. History *of delivery care use*, data available for *n* = 3682 as *n* = 45 women were first time mothers for whom history of delivery care was not applicable. *Assisted delivery mode*, data available for *n* = 3714 women. *n* = 11 had abortion outcomes and, therefore, delivery mode was not applicable while *n* = 2 had missing data

When variation of these factors was examined across districts, some differences were noted in education levels, prior service use, home visits by HEWs and the type of the closest health facility. Variation across districts in household wealth was notable, with 53% of Gomma residents falling within the least poor groups but only 15% belonging to these groups in Kersa district. Variability was also present between PHCUs, both within and across districts, and was the case for almost all potential explanatory factors. Using wealth as an example, the percentage of least poor households ranged from only 6% in Kusaye Beru PHCU (Kersa district) to 30% in Beshasha and 68% in Dhayi Kechene PHCUs (Gomma district) (*data not shown*).

### Global associations in service use identified by statistical regression models

Prior use of a maternal health service was the only factor that was strongly associated with current use of all three services (Table 2). Information source, household wealth and home visits by HEWs were found to be significantly associated with both ANC and delivery care but not PNC. Additionally, attitude towards delivery care, preparing for birth and type of nearby health facility, that were not hypothesized to be important for PNC use and, thus not included in the PNC models, were significantly associated with the other two services. Being involved in decision making, lower parity and the pregnancy being planned were important for ANC use while having an assisted delivery was significantly associated with PNC use. As hypothesized, having companion support was favourably associated with both delivery and postnatal care use. Awareness of danger signs was not a significant factor associated with delivery care use.

**Table 2:** Results from global random effects logistic regression analysis of antenatal, delivery and postnatal care use

Potential explanatory factor	Antenatal care (*n* = 3687)^a^		Delivery care (*n* = 3682)^a^		Postnatal care (*n* = 3708)^a^	
	OR (95% CI)		OR (95% CI)		OR (95% CI)	
**Individual factors**						
Education level						
None	–		Reference		Reference	
Primary/secondary/higher	–		0.77	(0.63,0.93)	1.09	(0.90, 1.33)
Pregnancy danger signs						
Not aware	Reference		–		–	
Aware	1.21	(1.02,1.44)	–		–	
Delivery danger signs						
Not aware	–		Reference		–	
Aware	–		1.22	(0.98, 1.51)	–	
Postpartum danger signs						
Not aware	–		–		Reference	
Aware	–		–		1.22	(1.02,1.46)
Nurse as information source						
Health information						
No	Reference		–		Reference	
Yes	2.08	(1.79,2.41)	–		0.94	(0.79, 1.13)
Delivery information						
No	–		Reference		–	
Yes	–		2.17	(1.82,2.58)	–	
Antenatal care use						
No prior use	Reference				–	
Prior use	1.87	(1.61,2.18)	–		–	
No use last pregnancy	–		Reference			
> = 4 last pregnancy	–		2.06	(1.73,2.44)		
Delivery care use						
No prior use	–		Reference		–	
Prior use	–		9.56	(7.67,11.92)	–	
No use last pregnancy	–		–		Reference	
Used for last pregnancy	–		–		15.35	(12.61,18.69)
Attitude towards delivery care						
Necessary for all	Reference		Reference		–	
Not necessary for all	0.51	(0.36, 0.71)	0.32	(0.22,0.47)	–	
Delivery mode						
Not assisted	–		–		Reference	
Assisted	–		–		2.95	(1.95,4.45)
**Household or inter-personal factors**						
Wealthiest household group						
No	Reference		Reference		Reference	
Yes	1.52	(1.30,1.79)	1.36	(1.12, 1.66)	1.20	(0.99,1.46)
Companion support						
Not available	–		Reference		Reference	
Available	–		2.75	(2.20,3.43)	1.64	(1.28,2.08)
Health-related decisions						
Not involved	Reference		–		Reference	
Involved	1.33	(1.13,1.57)	–		1.10	(0.91, 1.34)
Delivery site decisions						
Not involved	–		Reference		–	
Involved	–		0.83	(0.67, 1.02)	–	
Parity	0.92	(0.89,0.95)	1.04	(0.99,1.08)	0.98	(0.94, 1.02)
Pregnancy planned						
No	Reference		–		–	
Yes	1.42	(1.21,1.66)	–		–	
Birth preparedness						
Did not plan	Reference				–	
Planned for delivery	1.46	(1.24,1.71)	1.45	(1.20,1.74)	–	
**Health system factors**						
Home visit by HEW						
No	Reference		Reference			
Yes	1.32	(1.13, 1.55)	1.35	(1.13,1.62)	1.14	(0.95, 1.36)
Nearby health facility type/level						
Not hospital/health centre	Reference		Reference		–	
Hospital/health centre	1.66	(1.44,1.92)	1.98	(1.67,2.36)	–	

^a^Denominators indicate number of women for whom data was available for all candidate explanatory variables. Differences between models are reflective of differences in data available (Nurse as health information source *n* = 3721; history of ANC use *n* = 3688; history of delivery care use *n* = 3682 and delivery mode *n* = 3714)

Evidence of spatial autocorrelation in global model residuals was detected for all three services (*p* < 0.001) (results not shown).

### Local variation in associations of service use revealed by GWR models

**Fig. 2:**
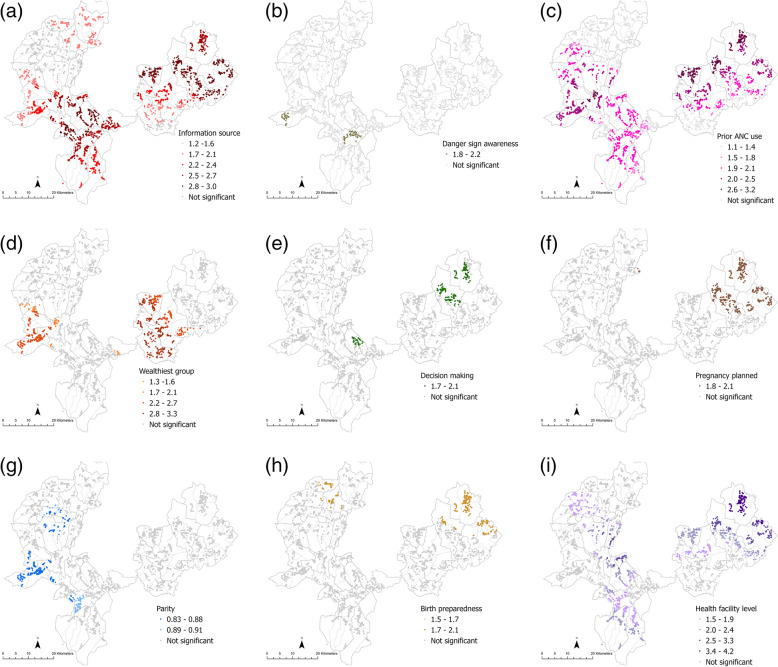
Local variation in relationships between ANC use and **a** information source **b** danger sign awareness **c** prior ANC use **d** wealthiest households **e** decision involvement **f** planned pregnancy **g** parity **h** birth preparedness **i** health facility level. Only magnitudes of statistically significant local odds ratios included in the legend

**Fig. 3:**
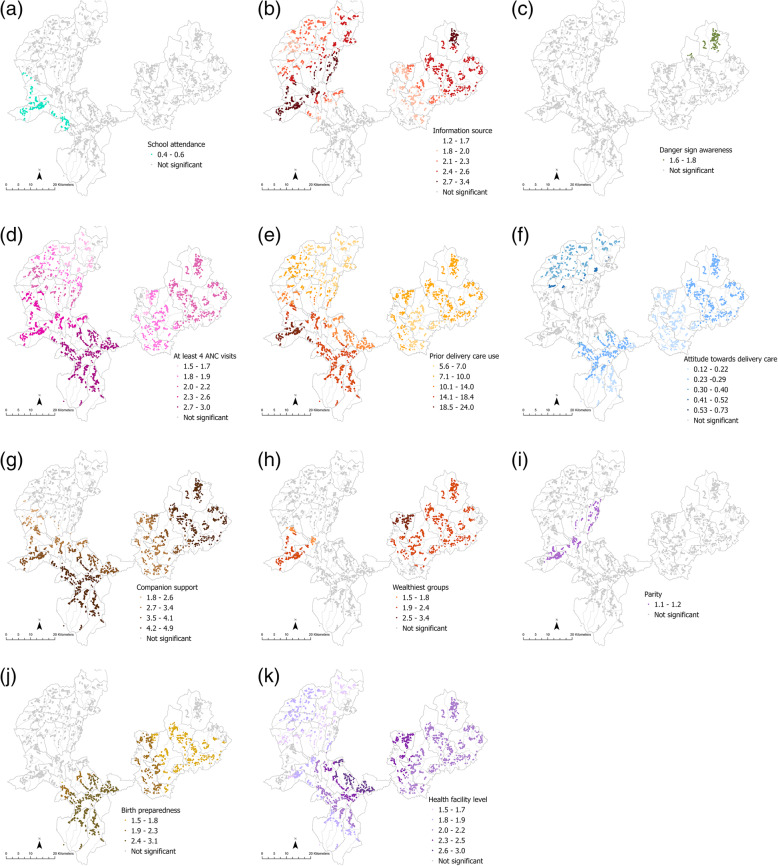
Local variation in relationships between delivery care use and **a** education **b** information source **c** danger sign awareness **d** at least 4 ANC visits **e** priory delivery care use **f** attitude towards delivery care **g** companion support **h** Wealthiest households **i** parity **j** birth preparedness **k** health facility level. Only magnitudes of statistically significant local odds ratios included in the legend

**Fig. 4:**
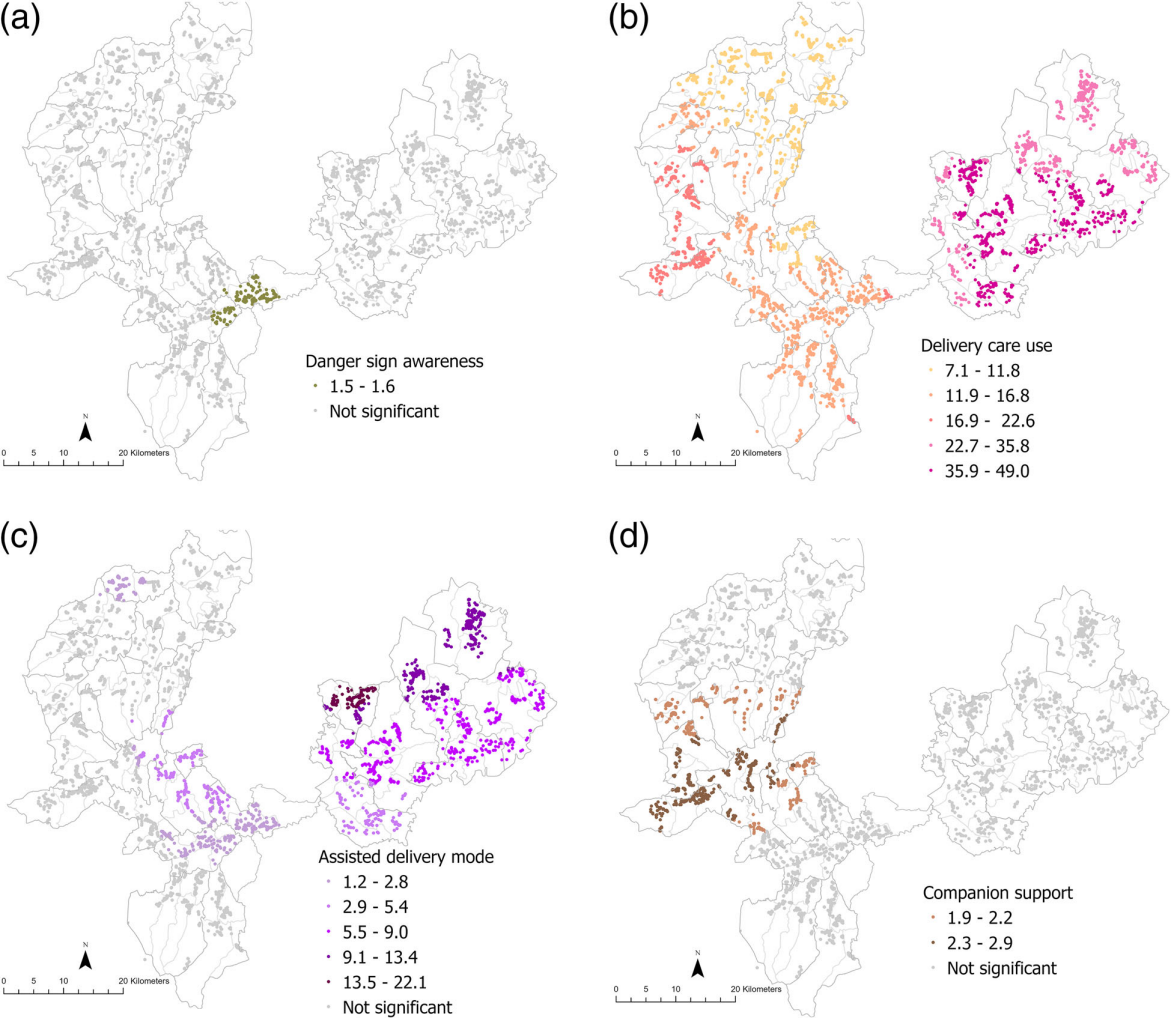
Local variation in relationships between PNC use and **a** danger sign awareness **b** delivery care use **c** assisted delivery more **d** companion support. Only magnitudes of statistically significant local odds ratios included in the legend

The panel of maps for ANC (Fig. 2a-i), delivery care (Fig. 3a-k) and PNC (Fig. 4a-d) depict estimates of local associations detected for each service and their respective explanatory variables. Variation in magnitude of local parameter estimates was visually apparent for all three service outcomes across most explanatory variables. However, whether or not local associations were statistically significant, the strength of the association, and at what scale the relationships appeared to vary, depended on the explanatory factor, service outcome and locality under consideration.

Comparison of results from the local GWR models and conventional global regression models revealed several things. Firstly, associations for some explanatory factors found to be statistically significant at the global level (Table 2) had widespread significant local associations as well, but differed in magnitude as illustrated by darker shades on maps. For instance, local estimates for ANC use and information source (Fig. 2a) or prior ANC use (Fig. 2c) were significant for households across most PHCUs in both Kersa and Seka Chekorsa districts as well as households in some Gomma districts *kebeles*. However, stronger associations between ANC use and information source could be seen in households in the northern PHCUs in Kersa district than those in the southern PHCUs (Fig. 2a). Similarly, local associations of prior ANC use among households in PHCUs along the north-western parts of Kersa district were of higher magnitude than households in Seka Chekorsa district PHCUs (Fig. 2c).

Statistically significant global associations, assumed to be relevant for the entire study area, were also found to have quite localized associations for certain factors when local model results were considered. Higher household wealth, for example, was most relevant for households in Kersa districts PHCUs and some households in *kebeles* in Geta Bake PHCU (Seka Chekorsa district) when it came to delivery care use (Fig. 3h). In the other areas, such as households in Gomma district, other factors such as prior service use (Fig. 3d or e) and attitudes towards care (Fig. 3f) appeared to be more relevant. Contrastingly in Kersa, a relatively poorer district than Gomma, in addition to higher wealth levels, having companion support was associated with delivery care use in the northern parts (Fig. 3g) but engaging in birth preparedness planning had significant associations with delivery care use in households in the southern *kebeles* (Fig. 3j).

The localities for which explanatory factors exerted an influence also differed depending on the service considered. Having nurses as an information source exhibited significant associations for both ANC and delivery care use but the areas where these associations were detected differed; for ANC use strong associations could be seen among households from PHCUs in the south-central portion of the study area coinciding with Setemma, Wokito and Bake Gudo PHCUs (Fig. 2a) while for delivery care no significant local estimates were detected for this factor in these areas (Fig. 3b). Similarly, while having companion support appeared to be important for delivery care use among households in most PHCUs in Kersa and Seka Chekorsa districts (Fig. 3g), it seemed to be relevant for fewer households concentrated mainly in *kebeles* from Geta Bake, Setemma and Kedemasa PHCUs for PNC use (Fig. 4d).

Interestingly, women’s involvement in decision making was found neither to be neither globally (Table 2) nor locally significant with respect to delivery care use and the test for spatial variability also did not find evidence of significant non-stationarity (results not shown). Finally, both global and local estimates for several explanatory factors for PNC use such as education, wealth, and parity were not statistically significant. However, spatial variability tests suggested that there was significant non-stationarity in relationships implying that the scale at which local associations were explored may be unsuitable (results not shown).

The Global Moran’s I test conducted on GWR residuals was significant for all three services, indicating that there was still some spatial autocorrelation present.

## Discussion

Conventional regression models identified a series of individual, interpersonal and health system factors as important for maternal health service use. Several factors such as danger sign awareness [37, 38], prior service use [39], wealth levels [40–42], parity [42–44] and whether or not a pregnancy was planned [37, 40, 45], have been reported by other studies investigating service use in Ethiopia. The use of GWR models, however, uncovered the existence of spatially varying associations between service use and explanatory factors suggesting that these factors may not be uniform in their influence on service use across the study area. Thus, GWRs can potentially facilitate the exploration of place effects in ways traditional regression models cannot. Statistical regression models often “control” for place by including population composition variables (such as the proportion of educated women in a village) as proxies for local context. However, as Tunstall and colleagues explain, this is unlikely to adequately capture the complex mechanisms that gave rise to these compositional differences to begin with [46].

Understanding how geographical and social contexts shape what factors have prominence in affecting service use is essential for effective policy formulation and implementation. It is also a key component in the design of responsive health systems, which are described as being able to “anticipate and adapt to changing needs” [47]. Indeed strategies to create responsive health systems include gathering empirical evidence about the needs of the community to adapt services accordingly [48]. In localities where wealth drives service use, ensuring out-of-pocket expenses are minimized could be effective in encouraging use; whereas deploying more community health workers to promote better danger sign awareness may be more relevant in places where use is highly dependent on awareness of risks. Providing contextually-tailored care has also been identified as a fundamental dimension of equity-oriented primary health care services [49]. Once again, this underscores the need to have a clear understanding of local influences that shape use to prevent one-size-fits-all policies from perpetuating structural inequities that marginalize populations by ignoring place-specific effects.

Non-stationarity in service use associations may also partially explain conflicting findings from different studies about what seems to be driving maternal health service use. Women’s involvement in decisions about service use, for instance, has been described to be particularly important in patriarchal or hierarchical contexts where women are not primary decision makers [50–53]. However, while some studies find women’s decision-making involvement to be significantly associated with service use [54–56], others have not [40, 57]. These studies originate from different districts and *kebeles* across Ethiopia and the results may partially be a consequence of this contextual diversity. In our study, we found involvement in health-related decision making to be a central factor affecting ANC use in very few *kebeles*. While these results do not downgrade the importance of women making their own decisions, they do raise the possibility that other factors with statistically significant local estimates may be more influential in some of these areas.

### Limitations

Characteristics of husbands, such as their education levels, were not included in the models due to concerns about selection bias and missing data. These factors may represent important explanatory variables missing from our model which could contribute to model misspecification and increase the likelihood of detecting spatial variation where none actually exists [27]. However, interpersonal and household variables such as decision making, social support and household wealth, were included which likely capture some of the important dimensions of husband influence. There is also an under-representation of health system factors (such as quality of care) and geographic factors (such as terrain) considered in our models. This reflects one of the limitations of GWR models where variables that do not have sufficient variability or that are common across large subsets of the data cannot be accommodated in the models.

A second limitation was related to the scale at which relationships between various explanatory factors and service outcomes were considered. Standard GWR models employ the use of a single bandwidth that is averaged across all independent variables in the model. This assumes that the relationships between each independent variable and the outcome operate at the same spatial scale [58]. Multiscale GWRs, which allow bandwidths to vary between explanatory variables, are currently not available for binary outcomes. However, bandwidth intervals can indicate the potential average spatial scales at which processes may be operating [59]. This may partially explain the spatial autocorrelation structure detected in the GWR residuals.

## Conclusions

The presence of significant spatial variation in the relationships between service use and corresponding individual, interpersonal/household and health system factors highlights the importance of using analytic methods suited to capturing this variation adequately. GWR models facilitate the detection and exploration of this variability thus contributing to a more nuanced understanding of context-specific effects. The use of multiscale GWR models, that support the examination of relationship differences at several spatial scales, could further enhance this understanding. Consideration of local variability in the relative importance of factors influencing service use is critical for the design of equity-oriented, responsive health systems and context-appropriate policy implementation.

## Supplementary Information

**Additional file 1.** Conceptual Model.

**Additional file 2.** Table of definitions of explanatory variables.

## Data Availability

Data used for this analysis will be provided upon reasonable request to Dr. Manisha Kulkarni (manisha.kulkarni@uottawa.ca).

## Abbreviations

AICc: Corrected Akaike Information Criterion
ANC: Antenatal care
CI: Confidence interval
GWR: Geographically weighted regression
HEW: Health extension worker
MWH: Maternity waiting home
PHCU: Primary health care unit
PNC: Postnatal care
VDP: Variance decomposition proportion
VIF: Variance inflation factor
