# Supplementary material for: Spatial variability in factors influencing maternal health service use in Jimma Zone, Ethiopia: a geographically-weighted regression analysis
Source: BMC Health Serv Res. 2021 May 12;21:454. doi: 10.1186/s12913-021-06379-3 (PMC8117568; doi:10.1186/s12913-021-06379-3)

Article title: Spatial variability in factors influencing maternal healthcare service use in Ethiopia: a geographically-weighted regression analysis

Corresponding author: Jaameeta Kurji
School of Epidemiology and Public Health
University of Ottawa
600 Peter Morand Crescent, Ottawa,
Ontario, K1G 5Z3, Canada
Email: jkurj022@uottawa.ca

Authors:
Jaameeta Kurji
School of Epidemiology and Public Health
University of Ottawa

Charles Thickstun
School of Epidemiology and Public Health
University of Ottawa

Gebeyehu Bulcha
Jimma Zone Health Office
Oromia Region, Ethiopia

Monica Taljaard
Ottawa Hospital Research Institute

Ziqi Li
Department of Geography & Geographic Information Science
University of Illinois

Manisha A. Kulkarni
School of Epidemiology and Public Health
University of Ottawa

Additional File 1: Conceptual Model

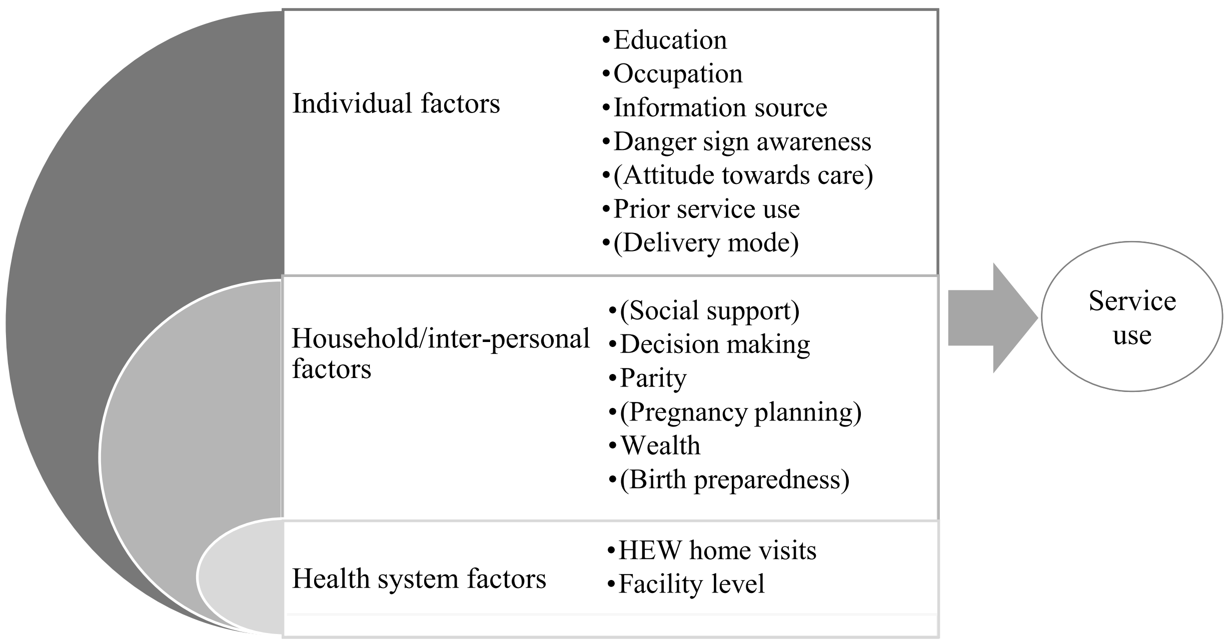

Supplement: Supplementary file 1 — Additional file 1. Conceptual Model. [file 12913_2021_6379_MOESM1_ESM.pdf]
