# Supplementary material for: Spatial variability in factors influencing maternal health service use in Jimma Zone, Ethiopia: a geographically-weighted regression analysis
Source: BMC Health Serv Res. 2021 May 12;21:454. doi: 10.1186/s12913-021-06379-3 (PMC8117568; doi:10.1186/s12913-021-06379-3)
Supplement: Supplementary file 2 — Additional file 2. Table of definitions of explanatory variables. [file 12913_2021_6379_MOESM2_ESM.pdf]

Article title: Spatial variability in factors influencing maternal healthcare service use in Ethiopia: a geographically-weighted regression analysis

Corresponding author: Jaameeta Kurji
School of Epidemiology and Public Health
University of Ottawa
600 Peter Morand Crescent, Ottawa,
Ontario, K1G 5Z3, Canada
Email: jkurj022@uottawa.ca

Authors:
Jaameeta Kurji
School of Epidemiology and Public Health
University of Ottawa

Charles Thickstun
School of Epidemiology and Public Health
University of Ottawa

Gebeyehu Bulcha
Jimma Zone Health Office
Oromia Region, Ethiopia

Monica Taljaard
Ottawa Hospital Research Institute

Ziqi Li
Department of Geography & Geographic Information Science
University of Illinois

Manisha A. Kulkarni
School of Epidemiology and Public Health
University of Ottawa

Additional File 2: Table of definitions of explanatory variables

Variable	Description
**Individual factors**	
Education	A binary variable created from women's responses about whether or not they had received any formal education at any level (primary, secondary or higher).
Information source	Two binary variables were created from multiple response variables indicating women's sources of information about (i) health or (ii) birth. Sources included nurses, HEWs, husbands, relatives, friends and others. Responses were classified into whether or not nurses (the most reliable source available) were listed as an information source. The nurses as sources for health-related information variable was used for the ANC and PNC models, while the nurses as sources of birth-related information source was used for the delivery care model
Danger sign awareness	Women were asked to list symptoms of serious health problems that can occur during pregnancy, birth or during the postpartum period. Binary variables (yes/no) were created to indicate whether or not women could name at least one danger sign (such as vaginal bleeding, severe headaches, blurred vision, convulsions, swollen face or hands, high fever, etc) during: (i) pregnancy (used for ANC model), (ii) birth (used for delivery care model) and, (iii) after birth (used for the PNC model)
Attitude towards care	A binary variable created from responses when women were asked if they agreed or disagreed with the statement that women with prior experience giving birth to a child did not need to deliver subsequent children at a health facility. Used for delivery care model.
Prior service use	Two binary variables were created indicating whether or not women reported: (i) ever using ANC services during past pregnancies (used for ANC model) and, (ii) ever delivering previous children at a health facility (used for delivery care model)
Delivery mode	This variable was hypothesized to be relevant only for PNC use. A binary variable was created to indicate whether or not the woman reported having an assisted delivery (caesarean section, vacuum or forceps extraction) during the birth of her last child.
**Interpersonal or household factors**	
Social support	Women were asked about several dimensions of social support. The dimension hypothesized to be most relevant for accessing services was having a companion to accompany women to the health facility. Women who indicated they had a companion available during pregnancy, labour and after delivery were classified as "Yes". Considered to be an inter-personal/household factor as women draw social support from various members of their social networks including husbands, family members, neighbours, friends, etc

Variable	Description
Involvement in decision making	Women were asked who was involved in decisions around the place of delivery and health-related decisions. Responses indicating their participation in the decision-making process (i.e., decision-maker included “self” or “jointly with husband”) were classified as “Involved” while all other responses (husband only, family member only) were classified as “Not involved”. The variable indicating involvement in health-related decisions was used for ANC and PNC models, while decision making around place of delivery was used for deliver care models.
Pregnancy planned	Women were asked if they had planned their last pregnancies. A binary variable (yes/no) was created from their responses.
Parity	A count variable indicating the total number of times women reporting having given birth to a child. Considered to be an inter-personal/household factor as the number of children a woman has is also influenced by her husband.
Wealth	An asset-based wealth index was created using principal components analysis on asset ownership (radio, television, mobile phone, motorbike, car/truck, livestock), presence of utilities (electricity and drinking water source), sanitation facilities, health insurance and dwelling construction materials. Scores were ranked and divided into quintiles. A binary variable was created for this analysis to indicate whether or not women belonged to the least poor households (quintiles four and five).
Birth preparedness	Women were asked if they did anything to prepare for the birth of their last child prior to delivery. Responses included items as such saving money, identifying means of transport or getting a referral for maternity waiting home use and were classified as “Yes”. Considered to be an inter-personal/household factor as certain planning dimensions such as organizing transport require input from husbands
**Health system factors**	
Home visit	A binary variable indicating whether or not a community-based health extension worker visited the woman’s home during the antenatal and postpartum period of her last child. Considered a health-system factor as HEWs represent the community-based segment of the health system structure.
Health facility type	A binary variable indicating whether or not women reported having a hospital or health centre near their home.
